# Nitrate in Maternal Drinking Water during Pregnancy and Measures of Male Fecundity in Adult Sons

**DOI:** 10.3390/ijerph192114428

**Published:** 2022-11-03

**Authors:** Pernille Jul Clemmensen, Nis Brix, Jörg Schullehner, Anne Gaml-Sørensen, Gunnar Toft, Sandra Søgaard Tøttenborg, Ninna Hinchely Ebdrup, Karin Sørig Hougaard, Birgitte Hansen, Torben Sigsgaard, Henrik Albert Kolstad, Jens Peter Ellekilde Bonde, Cecilia Høst Ramlau-Hansen

**Affiliations:** 1 Department of Public Health, Aarhus University, 8000 Aarhus, Denmark; 2 Department of Clinical Genetics, Aarhus University Hospital, 8200 Aarhus, Denmark; 3 Geological Survey of Denmark and Greenland, 8000 Aarhus, Denmark; 4 Danish Big Data Centre for Environment and Health (BERTHA), Aarhus University, 8000 Aarhus, Denmark; 5 Steno Diabetes Center Aarhus, Aarhus University Hospital, 8200 Aarhus, Denmark; 6 Department of Occupational and Environmental Medicine, Copenhagen University Hospital—Bispebjerg and Frederiksberg Hospital, 2400 Copenhagen, Denmark; 7 Department of Public Health, University of Copenhagen, 1014 Copenhagen, Denmark; 8 Fertility Clinic, The Regional Hospital in Horsens, 8700 Horsens, Denmark; 9 National Research Centre for the Working Environment, 2100 Copenhagen, Denmark; 10 Centre for Integrated Register-Based Research, Aarhus University (CIRRAU), 8000 Aarhus, Denmark; 11 Department of Occupational Medicine, Aarhus University Hospital, 8200 Aarhus, Denmark; 12 Department of Clinical Medicine, Aarhus University, 8200 Aarhus, Denmark

**Keywords:** prenatal exposure, semen quality, drinking water, nitrate, semen analysis, reproduction

## Abstract

Animal studies indicate deleterious effects of nitrate exposure on fecundity, but effects in humans are unknown, both for the prenatal and postnatal periods. We aimed to investigate if exposure to nitrate in maternal drinking water during the sensitive period of fetal life is associated with measures of fecundity in the adult sons. In a sub-analysis, the potential effects of nitrate exposure in adulthood were investigated. This cohort included 985 young adult men enrolled in The Fetal Programming of Semen Quality Cohort (FEPOS). Semen characteristics, testes volume and reproductive hormones were analyzed in relation to nitrate concentration in maternal drinking water, using a negative binomial regression model. The nitrate concentration in drinking water was obtained from monitoring data from Danish waterworks that were linked with the mothers’ residential address during pregnancy. The median nitrate concentration in maternal drinking water was 2 mg/L. At these low exposure levels, which are far below the World Health Organization’s (WHO) guideline value of 50 mg/L, we did not find indications of harmful effects of nitrate on the investigated measures of male fecundity.

## 1. Introduction

Around eight percent of Danish children are born after assisted reproductive techniques, making infertility a considerable public health concern. Male factor infertility is present in half of the infertility cases with a recognized cause [1]. Low semen quality contributes to male factor infertility and in several Western populations, semen quality is considered suboptimal [2]. Whether there has been a downward trend in semen quality is under debate [2,3]. Several environmental exposures are considered harmful for the male reproductive system [2,4,5]; however, the potential effects on male fecundity of nitrate exposure, a common contaminant in drinking water, are sparsely investigated [6,7]. Exposures during fetal life might define reproductive health in adulthood due to modifications of the developing reproductive system [5]. Findings from epidemiological studies suggest that exposure to nitrate during fetal life is capable of affecting the developing fetus negatively and nitrate exposure has been associated with increased risk of birth defects including central nervous system, eye and limb defects [6,8,9]. A suggested mechanism, by which nitrate can affect the developing fetus, is through the formation of teratogenic *N*-nitroso-compounds (NOCs). These are endogenously formed when nitrite, a reduced form of nitrate, reacts with dietary amines or amides and nitrosatable drugs [6,10]. Additionally, nitrate might affect the male reproductive system through endocrine disturbance, including impaired thyroid and steroid hormone production [6,11,12,13].

In addition to the fetal period, the period of post-pubertal spermatogenesis, may constitute a sensitive window for nitrate exposure [14,15]. Findings from experimental studies of sexually mature rats, mice and rabbits indicate a negative impact from prolonged nitrate exposure on the male reproductive system, observed as impaired semen characteristics, lower testes weights and testosterone levels, and altered activity of testicular enzymes. These effects were only observed at nitrate levels exceeding the expected levels in drinking water [16,17,18]; however, contrary to humans, rats and mice do not concentrate nitrate in saliva, and higher nitrate levels might therefore be needed in these species to induce the same effects as in humans [19]. Additionally, in rabbits, a difference in nitrate metabolism compared to humans has been indicated [20]. Furthermore, pubertal rats exposed to nitrosamines (a subgroup of NOCs) presented with lower levels of testosterone and FSH, and lower sperm count [21]. Nitrosamines are shown to cross the blood-testes barrier and form DNA-alkylating adducts in spermatogonia [22]. N7-methyldeoxyguanosine (N7-MedG) is a biomarker of exposure to DNA-alkylating agents and in a cohort study of couples attending a fertility clinic, men diagnosed with male factor infertility, had higher levels of N7-MedG in sperm compared to men not diagnosed with male factor infertility [23].

Given the described susceptible period of fetal life, the main aim in this study was to investigate if prenatal exposure to nitrate from maternal drinking water is associated with measures of male fecundity in the adult sons, namely impaired semen quality, lower testes volume and altered levels of reproductive hormones. As animal studies indicate a potential harmful effect of nitrate exposure towards the adult testes, we further, in a sub-analysis, investigated if nitrate exposure during the period of spermatogenesis, i.e., three months prior to delivery of a semen sample, was associated with measures of male fecundity.

## 2. Materials and Methods

Study design and population: This population-based cohort study was performed within The Fetal Programming of Semen Quality Cohort (FEPOS) [24]: a sub-cohort of the Danish National Birth Cohort (DNBC) [24,25]. The recruitment of the FEPOS cohort has previously been described in detail and is summarized in the following [24]. Written informed consent on all participants was obtained at enrolment. Eligible participants were young men aged 18 years and 9 months born of mothers in the DNBC who participated in the two computer-assisted telephone interviews during pregnancy around gestational weeks 16 and 31 [26] and had a blood sample taken during pregnancy. Further, the young men had to live near the clinics in Aarhus or Copenhagen. Of the 21,623 eligible sons, 5697 were invited to fill out a comprehensive online questionnaire and participate in a clinical examination that included collection of a semen and a blood sample and self-measurement of testes volume. In total, 1058 sons responded to the questionnaire and underwent the clinical examination from March 2017 until December 2019.

Estimation of nitrate exposure from drinking water: The Danish drinking water supply is based on groundwater, and almost 96% of Danish households are served by public waterworks where the drinking water quality, including nitrate concentration, is measured at certified laboratories at regular intervals, depending on the waterworks’ production volume. The measurement intervals vary between once every second year to several samples per month. The monitoring of unregulated private wells is limited. Therefore, private well users were excluded from the analyses [27]. Each waterworks’ distribution area has been mapped allowing for estimation of the yearly nitrate concentration in tap water at each Danish household [28]. If several samples were available for one year, the waterworks annual average was computed. For households located in water supply areas with more than one waterworks, annual averages were computed by weighing nitrate concentrations by the waterworks’ production volume. Of the households supplied by public supplies, 95% of the household-year combinations had at least one nitrate sample measured within the same year. For the remaining, we imputed nitrate estimates by linear interpolation, if a sample was available within three years to allow for smaller sampling frequencies in waterworks producing less than 100 m^3^ per day. For 2.4% of the combinations, a nitrate sample was available within one year, for additionally 1.6% within three years, while 1.1% of household-year combinations were classified as missing nitrate estimate as no sample was available within three years. The highest reported detection limit of nitrate in drinking water in our dataset was 1 mg/L and participants with nitrate levels below this limit are therefore included in the reference group (see below). The residential history of all Danish residents is registered in the Danish Civil Registration System (CRS), at a resolution of one day [29], enabling calculation of an individual-level exposure to nitrate from drinking water taking any movements in to account [27]. Nitrate exposure from drinking water (measured as NO_3_^−^, throughout the manuscript nitrate refers to NO_3_^−^) was assessed for the period of pregnancy (from pregnancy start to date of birth) in the main analysis, and for the three months prior to delivery of the semen sample at the clinical examination in a sub-analysis. Gestational age and date of birth was derived from the Danish Medical Birth Register (MBR) [30]. For the prenatal exposure period, we used the maternal residential history and, for the exposure period three months prior to the clinical examination, we used the residential history of the sons. We computed time-weighted averages to account for changes of residence and exposure periods covering more than one calendar year. Register-based information on residential history was available for the study population until April 2017 and on a postal code level in February 2020. Hence, for those men living in the same postal code in 2017 and in 2020, we derived the nitrate concentration assuming the men lived at the same residential address as in 2017. For those who had moved to a different postal code, we coded the nitrate concentration as missing.

The nitrate exposure from drinking water is in generally low in Denmark and this is reflected in the selection of exposure groups. The nitrate exposure was separated into three exposure groups; ≤2 mg/L (reference group), 2–5 mg/L, >5 mg/L inspired by a recent study on prenatal exposure to nitrate [31].

Assessment of semen characteristics, testes volume and reproductive hormones: The details on the blood and semen sample analyses have been reported previously [24]. The sons collected the semen sample either at home (*n* = 130) or at the clinics (*n* = 855). The analyses were performed in accordance with WHO’s recommendation for examination of human semen [32], and performed by two trained biomedical laboratory technicians. All semen samples were weighed to determine semen volume (1 g = 1 mL) and spillage was reported (yes/no). If there was spillage, the semen volume and total sperm count were coded as missing. Prior to analyses, the semen sample was liquefied in a 37 °C heating chamber at the clinics. Sperm concentration (million/mL) was counted manually, and total sperm count (million) was calculated by multiplication of semen volume and sperm concentration. Sperm motility (percentage of motile progressive, motile non-progressive and immotile sperm) was determined by counting of the numbers of motile progressive sperm, motile non-progressive sperm and immotile sperm. The evaluation of sperm morphology (percentage of sperm with normal morphology using the strict criteria) was performed at the Reproductive Medicine Centre, Skaane University Hospital, Sweden. The testes volumes were self-assessed by a Prader Orchidometer, which has been considered a valid method [33]. Non-fasting venous blood samples were collected during the clinical examination. The blood samples were stored at −80 °C until analysis in the autumn 2020. The limit of detection (LOD) for the hormones were: testosterone 0.12 nmol/L, estradiol 15 pmol/L, FSH and luteinizing hormone (LH) 0.1 IU/L and SHBG 0.350 nmol/L. A level of LOD/√2 was assigned to the few participants with values below the LODs (*n* ≤ 5 for FSH and LH and *n* = 84 for estradiol) [34].

Covariates: Information on highest educational level of parents, and maternal smoking in first trimester and pre-pregnancy body mass index (BMI) (<18.5; 18.5–25; 25–30; ≥30) was derived from the first DNBC pregnancy questionnaire, and information on maternal age at delivery came from the Danish Civil Registration System [29]. Information on numbers of neighbors residing within 250 m of the maternal residential address (≤500; 500–1000; 1000–2000; >2000) came from the residence database and were used as proxy for population density [35].

Information on the young men’s smoking status (Never, Occasional and Current smoker) and BMI (<18.5; 18.5–25; 25–30; ≥30) was derived from the FEPOS questionnaire.

Information on precision variables (variables with a strong association with the outcomes) was obtained from the clinical examination: abstinence time, place at semen sample collection, spillage, interval from ejaculation to analysis of semen sample, and time of the day for collection of blood sample. The subdivision of the covariates not already specified in the previous section is shown in Table 1.

**Figure 1:**
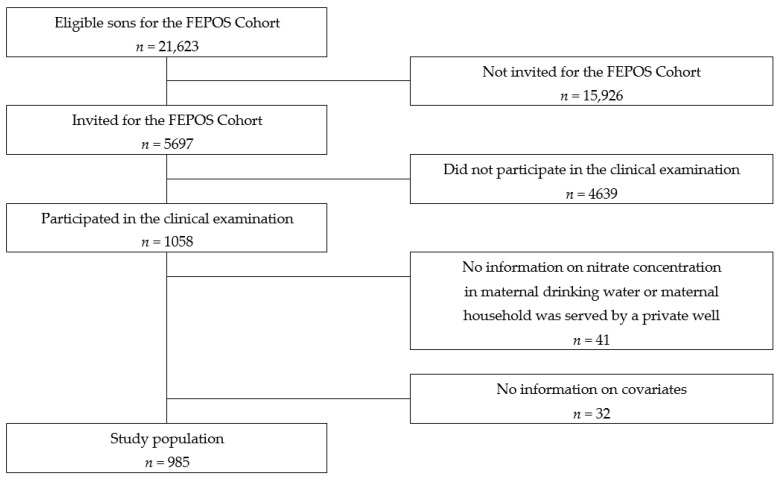
Flowchart of the recruitment of the study population.

Statistical analysis: For 41 of the sons, the nitrate water concentration at the maternal residential address during pregnancy was not available or the address was served by a private well, and 32 of the sons lacked information on one or more covariates; hence, the final study-population includes 985 sons (Figure 1).

**Figure 2:**
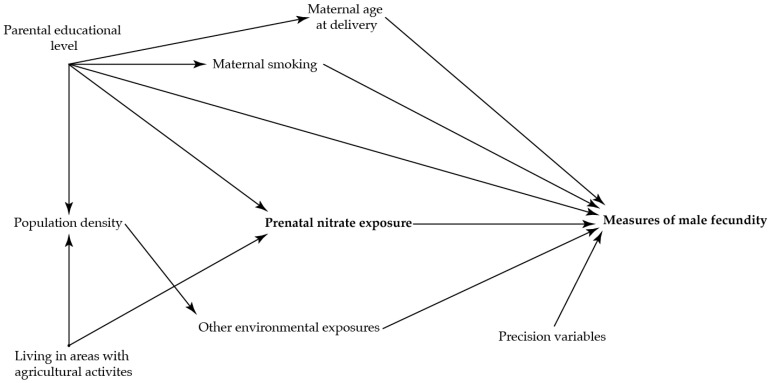
Directed acyclic graph illustrating the suggested associations between nitrate concentration in maternal drinking water and the reproductive health in the adult sons.

**Figure 3:**
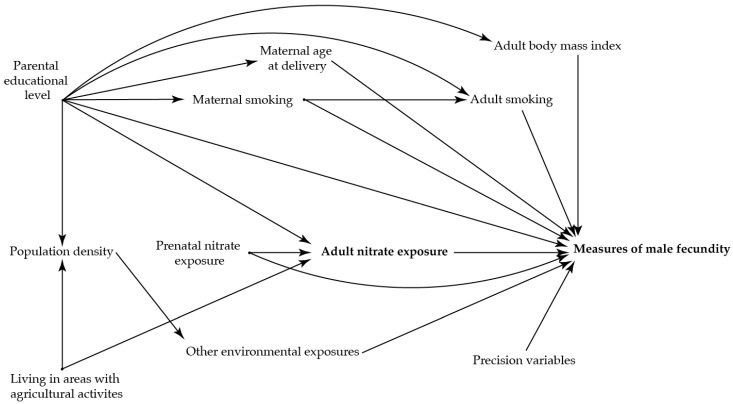
Directed acyclic graph illustrating the suggested associations between nitrate concentration in the adult sons’ drinking water three months prior to the clinical examination and the reproductive health in the adult sons.

To identify potential confounding variables, directed acyclic graphs were used prior to the analyses (Figure 2 and Figure 3)) [36]. The main analysis was adjusted for maternal age at delivery (as a second order polynomial), smoking in first trimester, and highest educational level of parents.

We performed three sub-analyses. In the first sub-analysis, a potential impact of nitrate in adulthood was investigated with the sons’ own exposure to nitrate from drinking water three months prior to the clinical examination (the period of spermatogenesis) [14]. This analysis was adjusted for the same covariates as the main model together with nitrate concentration in maternal drinking water in pregnancy (≤2 mg/L, 2–5 mg/L, >5 mg/L), the sons’ own smoking status and BMI.

In the second sub-analysis, we adjusted the main model for maternal BMI. As the evidence on the association between maternal BMI and the sons’ reproductive health is still sparse, we did not include BMI in the main analysis (Supplementary Table S1). In the third sub-analysis, we adjusted the main model for population density to account for other environmental exposures or socioeconomic factors related to place of residence (Supplementary Table S1). All analyses were further adjusted for precision variables, as given in the footnotes.

We used a multivariable negative binomial regression model to calculate differences in reproductive outcomes for each exposure category compared to the reference group exposed to ≤2 mg/L. Further, to investigate a potential linear association between nitrate and the reproductive health outcomes the categorical analysis was supplemented by analysis of nitrate concentration as a continuous variable investigating changes pr. 1 mg/L changes in nitrate concentration. The results are presented as relative differences in percent (ratio − 1) × 100% with 95% confidence intervals (CI). A potential non-linear relationship between nitrate and the reproductive health outcomes was also investigated, and the associations were visualized with an analysis of nitrate exposure as a restricted cubic spline variable with three knots (placed at the 10th, 50th and 90th percentile, as recommended by Harrell) [37].

Data were analyzed using Stata 15.0 software (Stata Corporation, College Station, TX, USA). To check the model assumptions, we compared the observed distribution of the reproductive outcomes with the model-based distributions using quantile-quantile plots. Then, standardized deviance residuals were plotted against the predicted mean values and the model fit was considered fair.

As not all 5697 invited sons participated in the FEPOS cohort (a participation rate of 19%), inverse probability weights were used to account for a potential selection bias [38]. The selection weights were calculated as the inverse probability of participation given maternal factors associated with participation and the exposure or the outcome (highest educational level of parents and maternal smoking, alcohol intake, pre-pregnancy BMI, region of invitation (Aarhus or Copenhagen), time-to-pregnancy and nitrate concentration in drinking water). Robust standard errors were used to account for clustering of siblings and the use of selection weights.

Due to local regulations (GDPR, Regulation (EU), 2016/679 of 25 May 2018) all percentiles, maximum and minimum values are reported as pseudo-numbers calculated as an average of the five surrounding values.

## 3. Results

The nitrate concentration in maternal drinking water during pregnancy was right-skewed with a median of 2 mg/L and a range from 0 mg/L to 31 mg/L. The Spearman correlation coefficient between nitrate exposure during pregnancy and the sons’ nitrate exposure during the three months prior to the clinical examination was 0.45 (*p* = 0.00).

Study participants exposed to a nitrate concentration of >5 mg/L in fetal life generally had parents with shorter education, mothers who more often smoked, did more often collect the semen sample at the clinic, had shorter time from delivery to the semen sample analysis and longer abstinence time compared to the reference group exposed to ≤2 mg/L (Table 1).

The baseline characteristics of semen quality, testes volume and reproductive hormones in relation to concentration of nitrate in maternal drinking water are presented in Table 2.

In the main analysis, the percentage of sperm cells with normal morphology was higher (22% [95% CI, 4;42]) and the percentage of non-progressive and immotile sperm cells (−9% [95% CI, −17;−1]) and FSH levels (−15% [95% CI, −27;−1]) were lower in the highest exposed group (>5 mg/L) compared to the reference group (≤2 mg/L) (Table 3). Further adjustment for maternal pre-pregnancy BMI or population density did not change the overall results (Supplementary Table S1). The analysis of nitrate concentration as a continuous variable showed no indication of a linear relationship between nitrate concentration in maternal drinking water and measures of male fecundity. In the adjusted spline plots (Supplementary Figure S1), the possible non-linear relationship of nitrate exposure in relation to measures of fecundity was illustrated. At higher exposure levels, semen volume and the percentage of non-progressive and immotile sperm cells were lower and estradiol levels were higher; though, the confidence intervals were wide.

In the sub-analysis on nitrate exposure in adulthood, no overall associations with the measures of fecundity were observed (Table 4).

## 4. Discussion

Findings in this large population-based cohort study did not indicate harmful effects of low-level prenatal exposure to nitrate from drinking water on the studied measures of fecundity in the adult sons. We did, however, observe higher levels of sperm cells with normal morphology, lower percentage of cells with compromised motility and lower FSH levels in the highest exposed group, but confidence intervals were wide, and the results were interpreted as chance findings or caused by residual confounding. The median nitrate exposure level observed in this study is far below the WHO guideline value of 50 mg/L nitrate in drinking water and comparable with nitrate levels observed in other studies performed in the Danish population [10,31,40]. Regardless of initiatives to lower the overall nitrate burden on the water systems in Europe, there are still areas with high exposures to nitrate [6]; hence, in the general Danish population, it is estimated that between 5.1% and 7.2% are exposed to concentrations above 25 mg/L [41]. Due to the low number of highly exposed participants in our study, we were unable to elucidate the potential effect of nitrate exposure at these higher exposure levels. Recent epidemiological studies of adverse birth outcomes and cancer suggest a negative impact of nitrate exposure also at lower levels than the guideline value [8,9,31,42,43,44,45]. A Danish study associated nitrate levels as low as 3.87 mg/L with increased risk of colorectal cancer [43], underlining the importance of studies on negative health effects also in low exposed populations.

Previously, epidemiological studies have associated prenatal exposure to other environmental exposures than nitrate with impaired male fecundity. Hence, a cohort study evaluating prenatal exposure to perfluorooctanoic acid (PFOA) found 34% lower sperm concentration (95% CI −58;5%) and 34% lower total sperm count (95% CI −62;12%) in the highest compared with the lowest exposed tertile, together with higher levels of LH and FSH [46]. Prenatal exposure to per- and polyfluoroalkyl substances were also investigated in the FEPOS cohort, where a one unit increase in exposure were associated with lowered sperm concentration (−8% (95% CI −16;−1)), total sperm count (−10% (95% CI −17;−2%)) and a higher proportion of non-progressive and immotile sperm cells (5% 95% CI (1;8%)) [47]. Another cohort study, investigated prenatal phthalate exposure and found lower testes and semen volume and higher FSH levels in the highest compared to the lowest exposed tertile [48]. A small decrease in sperm concentration might not affect fertility of the individual considerably; however, on population level, the observed effect sizes might be of concern. Some people might be more vulnerable towards environmental exposures than others due to heterogeneity of effects; taking nitrate as an example, differences between individuals in nitrate and NOC metabolism have been described [6].

Strengths and limitations: The large FEPOS cohort constitute a unique data source for study of the impact of prenatal exposure to nitrate on semen quality, testes volume and reproductive hormones in adulthood as the detailed information on these measures of fecundity in adulthood can be linked with the time specific monitoring of drinking water exposure at household level in Denmark. This mapping allows us to assess the drinking water exposure during two important time windows.

The participation rate in the FEPOS cohort had a low response rate of 19% [24]. To account for potential selection bias, we applied selection-weights. Since the participating men were young, we consider that their reproductive health would not have affected their participation in FEPOS as they are most likely still unaware of their reproductive potential. Further, the median nitrate concentration in maternal drinking water was similar in participants and non-participants; thus, the exposure was not associated with participation. However, in the analysis investigating the young men’s nitrate exposure during the three months prior to the clinical examination, there might be an association between nitrate exposure and participation as nitrate concentration depends on geography and sons living far from the clinics could be less prone to participate. This will, however, not lead to selection bias if the outcome is not associated with participation. In conclusion, we do not consider selection bias to be a major source of bias in this study.

Estimation of nitrate exposure at household level might include uncertainties. We did not measure the actual intake of nitrate from tap water. In Denmark, the intake of bottled water is low [8], and we therefore used the nitrate concentration in drinking water at the residential address as a proxy for the intake. When no measurement within the year of pregnancy was available, we used measurements taken up to three years from the year of pregnancy. This is considered valid due to low seasonal variation and low short term variation in nitrate concentration in drinking water [49]. The potential exposure misclassification is believed to be random across the nitrate concentration levels and unrelated to the outcomes and will possibly bias the estimates towards no association.

Testes volume was self-assessed and as reported previously there might be a small risk for underestimation [33]. Potential measurement errors of the outcome are probably also non-differential, as the sons were unaware of their exposure status at the time of the clinical examination [24].

One of the suggested mechanisms of a harmful effect of nitrate is formation of teratogenic NOCs. This formation can be inhibited by intake of antioxidants from supplements and diet and stimulated by intake of nitrosatable drugs [6]. In this study, we did not include information on dietary nitrate or stimulators/inhibitors of NOC formation. In a recent study from the DNBC (unpublished results), nitrate intake from diet was not associated with nitrate concentration in maternal drinking water, and we did not include nitrate in diet as a confounding variable in this study. We suggest future studies to cover this research area.

Sons included in the FEPOS cohort had to live near the clinics in Aarhus and Copenhagen where the nitrate concentration is, in general, low; however, their mothers could live in all parts of Denmark during pregnancy. If all the highly exposed study participants lived in the same areas there is a risk of residual confounding from shared socioeconomic factors and other environmental exposures related to place of living. To account for this, we adjusted our analyses for several confounding variables and in a sensitivity analysis, we adjusted for numbers of neighbors living within 250 m of the residential address.

## 5. Conclusions

To our knowledge, this is the first study in humans to investigate if prenatal exposure to nitrate from drinking water is associated with measures of fecundity in adulthood. We used unique household-level exposure information on nitrate concentration in maternal drinking water. The study population generally had low exposure to nitrate, and we did not observe an overall association between nitrate exposure at these low exposure levels and the measures of fecundity. We suggest replicating the study in a population with more highly exposed individuals to elucidate if nitrate levels below the WHO guideline value can be considered safe with regard to the male reproductive system.

## Figures and Tables

**Table 1:** Baseline characteristics of the study population in relation to nitrate exposure category.

	Nitrate Concentration in Maternal Drinking Water (mg/L)		
	≤2 (*n* = 548)	2–5 (*n* = 346)	>5 (*n* = 91)
**Maternal characteristics**			
Age at delivery in years			
Mean (±SD)	31 (4)	30 (4)	30 (4)
Highest social class of parents, *n* (%)			
High grade professional	203 (37.0)	116 (33.5)	23 (25.3)
Low grade professional	189 (34.5)	102 (29.5)	32 (35.2)
Skilled worker or unskilled worker	122 (22.3)	117 (33.8)	≥31 (~34.0)
Student or economically inactive	34 (6.2)	11 (3.2)	≤5 (~5.5) ^a^
Daily number of cigarettes in 1st trimester, *n* (%)			
Non-smoker	436 (79.6)	259 (74.9)	≥60 (~65.9)
≤10 cigarettes/day	97 (17.7)	68 (19.7)	26 (28.6)
>10 cigarettes/day	15 (2.7)	19 (5.5)	≤5 (~5.5) ^a^
**Characteristics of the clinical examination**			
Place of semen sample collection, *n* (%)			
At home	82 (15.0)	43 (12.4)	5 (5.5)
At the clinic	466 (85.0)	303 (87.6)	86 (94.5)
Interval between ejaculation and time of analysis in minutes, *n* (%)			
≤60	451 (82.3)	292 (84.4)	81 (89.0)
>60	97 (17.7)	54 (15.6)	10 (11.0)
Abstinence time in days, *n* (%)			
≤2	301 (54.9)	199 (57.5)	45 (49.5)
2–3	138 (25.2)	88 (25.4)	28 (30.8)
>3	109 (19.9)	59 (17.1)	18 (19.8)
Spillage, *n* (%)			
No	458 (83.6)	280 (80.9)	76 (83.5)
Yes	90 (16.4)	66 (19.1)	15 (16.5)
Time at blood sample, *n* (%)			
Morning <12 PM	207 (37.8)	110 (31.8)	38 (41.8)
Afternoon 12–18 PM	289 (52.7)	192 (55.5)	43 (47.3)
Evening >18 PM	52 (9.5)	44 (12.7)	10 (11.0)

Abbreviations: *n*, numbers; mg, milligram; L, liter; PM, post meridiem. a: cells with values below five are reported as ≤5 and a number in another cell in the column is modified to avoid reporting of numbers below 5 in accordance with local regulations (GDPR, Regulation (EU), 2016/679 of 25 May 2018).

**Table 2:** Baseline information on reproductive outcomes according to nitrate concentration in maternal drinking water.

Nitrate Concentration in Drinking Water (mg/L)			
	≤2 (*n* = 548)	2– 5 (*n* = 346)	> 5 (*n* = 91)
Semen quality characteristics ^a^			
Volume (mL) ^b^	3 (1;5)	3 (1;4)	3 (1;5)
Concentration (million/mL)	40 (8;115)	36 (6;102)	42 (7117)
Total sperm count (million) ^b^	102 (19;339)	96 (17;310)	122 (14;410)
Normal morphology (%) ^c^	6 (1;13)	6 (1;13)	8 (1;15)
Motility, non-progressive and immotile (%) ^c^	37 (19;60)	37 (21;61)	34 (19;55)
Testes volume ^a^			
Average volume (mL)	15 (9;23)	15 (9;25)	15 (9;23)
Reproductive hormones ^a^			
Estradiol (pmol/L)	53 (18;96)	52 (12;90)	54 (28;107)
Follicle stimulating hormone (IU/L)	4 (2;7)	4 (2;7)	3 (1;7)
Luteninizing hormone (IU/L)	5 (3;8)	5 (3;8)	5 (3;9)
Sex hormone binding globulin (nmol/L)	33 (21;51)	32 (20;52)	31 (19;47)
Testosterone (nmol/L)	18 (12;26)	18 (11;26)	18 (12;25)
Free androgen index (%)	56 (36;79)	54 (34;85)	57 (40;91)

Abbreviations: mL, milliliter; nmol, nanomolar; IU, international units; mg, milligramme; pmol, picomole. Lower reference values: Semen volume (1.5 mL). Concentration (15 million/mL). Total sperm count (39 million). Normal morphology (4%). Motility, progressive (32%) [32]. Testes volume (15 mL) [39]. ^a^: 50th percentile (10th; 90th percentiles). Reported as pseudo percentiles in accordance with local regulations (GDPR, Regulation (EU), 2016/679 of 25 May 2018). ^b^: excluding samples with spillage. ^c^: excluding samples from participants with azoospermia.

**Table 3:** Relative difference in percent in semen quality characteristics, testes volume and reproductive hormones in adult sons in relation to nitrate concentration in maternal drinking water.

	Nitrate mg/L	*n* ^a^	Crude	Adjusted ^b^ (95% CI)
**Semen quality characteristics**				
Volume (mL) ^c^		>790		
	≤2		ref	ref
	2–5		−3%	0% (−7;7)
	>5		7%	8% (−4;22)
	Per 1 mg/L nitrate		0%	0% (−1;1)
Concentration (million/mL) ^d^		>950		
	≤2		ref	ref
	2–5		−10%	−6% (−16;6)
	>5		−4%	0% (−17;19)
	Per 1 mg/L nitrate		−1%	0% (−2;1)
Total sperm count (million) ^c^		>790		
	≤2		ref	ref
	2–5		−6%	2% (−10;16)
	>5		16%	16% (−4;39)
	Per 1 mg/L nitrate		0%	0% (−1;2)
Normal morphology (%) ^e^		>930		
	≤2		ref	ref
	2–5		−3%	−2% (−11;8)
	>5		19%	22% (4;42)
	Per 1 mg/L nitrate		1%	1% (−1;2)
Motility, non-pregressive and immotile (%) ^e,f^		>935		
	≤2		ref	ref
	2–5		1%	0% (−5;6)
	>5		−8%	−9% (−17;−1)
	Per 1 mg/L nitrate		−1%	−1% (−2;0)
**Testes volume**				
Average volume (mL) ^g^		>950		
	≤2		ref	ref
	2–5		1%	1% (−3;6)
	>5		1%	1% (−7;9)
	Per 1 mg/L nitrate		0%	0% (−1;1)
**Reproductive hormones**				
Estradiol (pmol/L) ^h^		>950		
	≤2		ref	ref
	2–5		−5%	−4% (−11;3)
	>5		9%	8% (−3;22)
	Per 1 mg/L nitrate		1%	1% (0;2)
Follicle stimulating hormone (IU/L) ^h^		>950		
	≤2		ref	ref
	2–5		−1%	−1% (−11;10)
	>5		−15%	−15% (−27;−1)
	Per 1 mg/L nitrate		−1%	−1% (−2%;1)
Luteninizing hormone (IU/L) ^h^		>950		
	≤2		ref	ref
	2–5		−3%	−2% (−8;5)
	>5		−8%	−8% (−18;2)
	Per 1 mg/L nitrate		0%	0% (−1;1)
Sex hormone binding globulin (nmol/L) ^h^		>950		
	≤2		ref	ref
	2–5		−2%	−1% (−6;4)
	>5		−9%	−8% (−16;0)
	Per 1 mg/L nitrate		0%	0% (−1;0)
Testosterone (nmol/L) ^h^		>950		
	≤2		ref	ref
	2–5		−3%	−1% (−6;3)
	>5		−3%	−3% (−9;3)
	Per 1 mg/L nitrate		0%	0% (0;1)
Free androgen index (%) ^h^		>950		
	≤2		ref	ref
	2–5		3%	3% (−4;10)
	>5		6%	3% (−5;11)
	Per 1 mg/L nitrate		0%	0% (0;1)

Abbreviations: mg, milligramme, mL, milliliter; L, litre; nmol, nanomole, pmol, picomole; IU, international units; *n*, numbers; ref, reference. ^a^: rounded down to the nearest fifth due to local regulations. ^b^: adjusted for maternal age at delivery, maternal smoking during 1st trimester, highest educational level of parents. ^c^: excluding samples with spillage and further adjusted for abstinence time and place at semen sample collection; at home/at the clinic. ^d^: further adjusted for spillage, abstinence time and place at semen sample collection; at home/at the clinic. ^e^: excluding samples from participants with azoospermia. Further adjusted for spillage, abstinence time and place at semen sample collection; at home/at the clinic. ^f^: further adjusted for interval from ejaculation to analyses of semen sample. ^g^: further adjusted for abstinence time. ^h^: further adjusted for time at the day for collection of blood sample.

**Table 4:** Relative difference in percent in semen quality characteristics, testes volume and reproductive hormone levels in relation to nitrate concentration in the sons’ drinking water three months prior to the clinical examination.

	Nitrate mg/L	*n* ^a^	Adjusted ^b^ (95% CI)
**Semen quality characteristics**			
Volume (mL) ^c^		>625	
	≤2		ref
	2–5		2% (−7;12)
	>5		2% (−12;18)
	Per 1 mg/L nitrate		0% (−1;1)
Concentration (million/mL) ^d^		>765	
	≤2		ref
	2–5		9% (−6;26)
	>5		−17% (−36;8)
	Per 1 mg/L nitrate		−1% (−3;1)
Total sperm count (million) ^c^		>630	
	≤2		ref
	2–5		6% (−10;24)
	>5		−10% (−33;22)
	Per 1 mg/L nitrate		−1% (−3;1)
Normal morphology (%) ^e^		>745	
	≤2		ref
	2–5		11% (−1;25)
	>5		7% (−16;37)
	Per 1 mg/L nitrate		1% (−1;3)
Motility, non-progressive and immotile (%) ^e,f^		>750	
	≤2		ref
	2–5		−9% (−16;−2)
	>5		−9% (−21;5)
	Per 1 mg/L nitrate		−1% (−2;0)
**Testes volume**			
Average volume (mL) ^g^		>760	
	≤2		ref
	2–5		1% (−5;7)
	>5		5% (−5;16)
	Per 1 mg/L nitrate		0% (−1;1)
**Reproductive hormones**			
Estradiol (pmol/L) ^h^		>765	
	≤2		ref
	2–5		−7% (−16;4)
	>5		3% (−13;20)
	Per 1 mg/L nitrate		0% (−1;1)
Follicle stimulating hormone (IU/L) ^h^		>760	
	≤2		ref
	2–5		0% (−10;11)
	>5		−5% (−27;24)
	Per 1 mg/L nitrate		−1% (−2;1)
Luteninizing hormone (IU/L) ^h^		>760	
	≤1		ref
	2–5		1% (−6;8)
	>5		−3% (−19;15)
	Per 1 mg/L nitrate		−1% (−2;0)
Sex hormone binding globulin (nmol/L) ^h^		>760	
	≤2		ref
	2–5		4% (−2;11)
	>5		5% (−5;17)
	Per 1 mg/L nitrate		0% (0;1)
Testosterone (nmol/L) ^h^		>765	
	≤1		ref
	2–5		4% (−1;10)
	>5		−1% (−9,8)
	Per 1 mg/L nitrate		0% (−1;1)
Free androgen index (%) ^h^		>760	
	≤2		ref
	2–5		6% (−2;16)
	>5		−6% (−15;4)
	Per 1 mg/L nitrate		0% (−1;0)

Abbreviations: mg, milligram, mL, milliliter; L, liter; nmol, nanomole, pmol, picomole; IU, international units; *n*, numbers; ref, reference. ^a^: rounded down to the nearest fifth due to local regulations. ^b^: adjusted for maternal age at delivery, maternal smoking during 1st trimester, highest educational level of parents, maternal nitrate exposure during pregnancy, the sons’ smoking status and the sons BMI. ^c^: excluding samples with spillage and further adjusted for abstinence time and place at semen sample collection; at home/at the clinic. ^d^: further adjusted for spillage, abstinence time and place at semen sample collection; at home/at the clinic. ^e^: excluding samples from participants with azoospermia. Further adjusted for spillage, abstinence time and place at semen sample collection; at home/at the clinic. ^f^: further adjusted for interval from ejaculation to analyses of semen sample. ^g^: further adjusted for abstinence time. ^h^: further adjusted for time at the day for collection of blood sample.

## Data Availability

Data from the Danish National Birth Cohort can be accessed after permission from the DNBC Steering Committee. Information on the application procedure for data access to the DNBC is available at the website: https://www.dnbc.dk/access-to-dnbc-data (accessed on 27 October 2022).

## Supplementary Materials

The following supporting information can be downloaded at: https://www.mdpi.com/article/10.3390/ijerph192114428/s1, Figure S1: Spline plots with three knots (10th, 50th, 90th percentiles) and 95% confidence intervals showing the association between prenatal exposure to nitrate and semen characteristics, testes volume and reproductive hormones. Table S1: Further adjustment of the main-model for maternal pre-pregnancy BMI and population density. Relative difference in percent in semen quality characteristics, testes volume and reproductive hormones in adult sons in relation to nitrate concentration in maternal drinking water.

## Abbreviations

DNBC: The Danish National Birth Cohort; NOCs, *N*-nitroso compounds; FEPOS, Fetal Programming of Semen Quality cohort; CI, confidence interval; HPG, hypothalamic-pituitary-gonadal; MBR, the Danish Medical Birth Register; BMI, body mass index; FSH, follicle stimulating hormone; LH, luteinizing hormone; LOD, limit of detection; CRS, the Danish Civil Registration System.
