# Supplementary material for: Nitrate in Maternal Drinking Water during Pregnancy and Measures of Male Fecundity in Adult Sons
Source: Int J Environ Res Public Health. 2022 Nov 3;19(21):14428. doi: 10.3390/ijerph192114428 (PMC9656746; doi:10.3390/ijerph192114428)
Supplement: Supplementary file 1 [file ijerph-19-14428-s001.zip › ijerph-1971110-supplementary.pdf]

Supplementary Materials

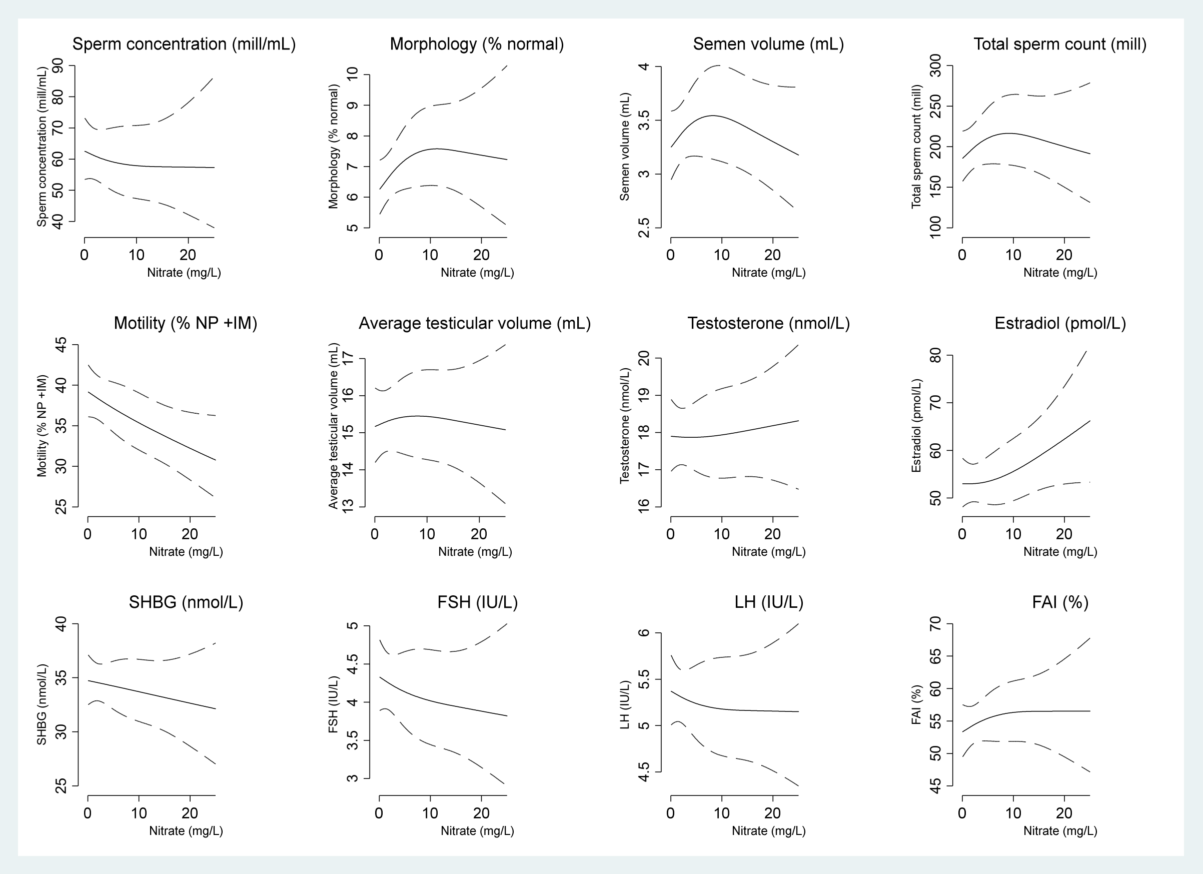

Figure S1. Spline plots with three knots (10th, 50th, 90th percentiles) and 95% confidence intervals showing the association between prenatal exposure to nitrate and semen characteristics, testes volume and reproductive hormones.

The spline plots are made for a reference son who had an abstinence time of 2-3 days, delivered the semen sample at the clinic, reported no spillage, had the semen sample analyzed <60 minutes from delivery and had the blood test in the afternoon. The reference sons' mother were a non-smoker during pregnancy and aged 30 years at delivery. The highest educational level of the reference sons' parents were high-grade professional.

Table S1. Further adjustment of the main model for maternal pre-pregnancy BMI and population density. Relative difference in percent in semen quality characteristics, testes volume and reproductive hormones in adult sons in relation to nitrate concentration in maternal drinking water.

**Semen quality characteristics**	Nitrate mg/L	n[a]	Crude	Adjusted for maternal pre-pregnancy BMI[b,c]	Adjusted for population density[b,d]
Volume (mL)[e]		>790			
	≤2		ref	ref	ref
	2-≤5		-3%	0% (-7;7)	0% (-7;7)
	>5		7%	8% (-4;22)	7% (-5;20)
	Per 1 mg/L nitrate		0%	0% (-1;1)	0% (-1;1)
Concentration (million/mL)[f]		>950			
	≤2		ref	ref	ref
	2-≤5		-10%	-6% (-16;6)	-6% (-16;6)
	>5		-4%	0% (-16;20)	-1% (-17;19)
	Per 1 mg/L nitrate		-1%	0% (-2;1)	0% (-2;1)
Total sperm count (million)[e]		>790			
	≤2		ref	ref	ref
	2-≤5		-6%	2% (-10;16)	2% (-10;16)
	>5		16%	16% (-3;40)	15% (-4;39)
	Per 1 mg/L nitrate		0%	0% (-1;2)	0% (-1;2)
Normal morphology (%)[g]		>930			
	≤2		ref	ref	ref
	2-≤5		-3%	-2% (-11;8)	-2% (-12;8)
	>5		19%	21% (4;42)	24% (6;46)
	Per 1 mg/L nitrate		1%	1% (-1;2)	1% (-1;2)
Motility, non-progressive and immotile (%)[g,h]		>935			
	≤2		ref	ref	ref
	2-≤5		1%	0% (-5;7)	1% (-5;6)
	>5		-8%	-9% (-17;-1)	-10% (-17;-1)
	Per 1 mg/L nitrate		-1%	-1% (-2;-0)	-1% (-2;-0)
**Testes volume**					
Average volume (mL)[i]		>950			
	≤2		ref	ref	ref
	2-≤5		1%	1% (-3;6)	2% (-3;7)
	>5		1%	1% (-6;9)	0% (-7;8)
	Per 1 mg/L nitrate		0%	0% (-1;1)	0% (-1;1)
**Reproductive hormones**					
Estradiol (pmol/L)[j]		>950			
	≤2		ref	ref	ref
	2-≤5		-5%	-4% (-11;3)	-4% (-11;3)
	>5		9%	8% (-4;21)	9% (-3;23)
	Per 1 mg/L nitrate		1%	1% (0;2)	1% (0;2)

Follicle stimulating hormone (IU/L)[j]		>950			
	≤2		ref	ref	ref
	2-≤5		-1%	-1% (-11;10)	-1% (-12;11)
	>5		-15%	-15% (-26;-1)	-14% (-26;-2)
	Per 1 mg/L nitrate		-1%	-1% (-2%;1)	0% (-2%;1)
Luteninizing hormone (IU/L)[j]		>950			
	≤2		ref	ref	ref
	2-≤5		-3%	-2% (-8;5)	-2% (-8;5)
	>5		-8%	-8% (-18;2)	-7% (-17;3)
	Per 1 mg/L nitrate		0%	0% (-1;0)	0% (-1;1)
Sex hormone binding globulin (nmol/L)[j]		>950			
	≤2		ref	ref	ref
	2-≤5		-2%	-1% (-6;4)	-1% (-6;4)
	>5		-9%	-8% (-15;0)	-8% (-16;0)
	Per 1 mg/L nitrate		0%	0% (-1;0)	0% (-1;0)
Testosterone (nmol/L)[j]		>950			
	≤2		ref	ref	ref
	2-≤5		-3%	-2% (-6;3)	-2% (-6;3)
	>5		-3%	-3% (-9;3)	-2% (-8;4)
	Per 1 mg/L nitrate		0%	0% (0;1)	0% (0;1)
Free androgen index (%)[j]		>950			
	≤2		ref	ref	ref
	2-≤5		3%	2% (-4;8)	2% (-4;9)
	>5		6%	2% (-5;11)	4% (-3;13)
	Per 1 mg/L nitrate		0%	0% (0;1)	0% (0;1)

a: rounded down to the nearest fifth due to local regulations. b: adjusted for maternal age at delivery, maternal smoking during 1st trimester and highest educational level of parents. c: further adjusted for maternal pre-pregnancy body mass index. d: further adjusted for population density (numbers of neighbors residing within 250 meters of the maternal residential address). e: excluding samples with spillage and further adjusted for abstinence time and place at semen sample collection; at home/at the clinic. f: further adjusted for spillage, abstinence time and place at semen sample collection; at home/at the clinic. g: excluding samples from participants with azoospermia. Further adjusted for spillage, abstinence time and place at semen sample collection; at home/at the clinic. h: further adjusted for interval from ejaculation to analyses of semen sample. i: further adjusted for abstinence time. j: further adjusted for time at the day for collection of blood sample
